# Cancer immunotherapy by immunosuppression

**DOI:** 10.1186/1742-4682-7-45

**Published:** 2010-12-15

**Authors:** Richmond T Prehn, Liisa M Prehn

**Affiliations:** 1 Department of Pathology, University of Washington, 5433 South Hudson St. Seattle, WA 98118, USA

## Abstract

We have previously suggested that the stimulatory effect of a weak immune reaction on tumor growth may be necessary for the growth of incipient tumors. In the present paper, we enlarge upon and extend that idea by collecting evidence in the literature bearing upon this **new hypothesis that a growing cancer**, **whether in man or mouse, is throughout its lifespan, probably growing and progressing because of continued immune stimulation by a weak immune reaction**. We also suggest that prolonged immunosuppression might interfere with progression and thus be an aid to therapy. While most of the considerable evidence that supports the hypothesis comes from observations of experimental mouse tumors, there is suggestive evidence that human tumors may behave in much the same way, and as far as we can ascertain, there is no present evidence that necessarily refutes the hypothesis.

## Background

There is, in our opinion, much still to be derived from the study of immunity to mouse cancers that is probably relevant to the human disease.

We have previously suggested, based largely upon mouse studies, that *incipient *cancers are probably stimulated to grow by a stimulatory immune reaction. We think the evidence suggests that a new expanded hypothesis is tenable: **any tumor that continues to grow is probably being *continuously *immunologically stimulated by a low level of immunity**. We will also discuss the therapeutic implications of this new hypothesis. Almost the entire literature seems currently to be predicated upon the assumption that the immune response is "surveying" and inhibiting cancer, if indeed it does anything. Our new hypothesis admits the persisting possibility of a cure by sufficiently raising the level of the immune reaction via immunizations of various types, but recognizes that this has not yet met with unmitigated success and that there may be an alternative. We were startled to see where the data logically leads and chagrined that we did not appreciate the possibility of this new hypothesis years ago.

For present purposes we define tumor immunity as any alteration, induced in the host by prior exposure to a tumor, that causes a change, **either positive or negative**, in the growth characteristics of that tumor when subsequently implanted in that host.

The following four conclusions, which we will expound upon in detail in subsequent paragraphs, are probably valid for both mouse and human cancers and each is necessary if this new hypothesis is to be regarded seriously:

1). The first conclusion is that very weak immune reactions tend to be stimulatory to tumor growth while quantitatively larger amounts of the same immune reactants may be inhibitory.

2). A second conclusion is that even the most highly immunogenic of mouse tumors, produced for example, by relatively large dosages of 3-methylcholanthrene (MCA), exhibit little or no detectable immunizing ability when tested in the very mouse in which the challenge tumor had originated.

3). Thirdly, those rodent tumors that occur sporadically without known cause have been almost invariably judged to be non-immunogenic [1], a judgment we suggest, on the basis of the available meager evidence, is erroneous.

4). Lastly, there is much evidence that immunity influences progression in both mouse and human tumors.

If these four conditions are met there seems to be no currently valid obstacle to the new hypothesis that **most (all or many) growing tumors are driven at least in part by an immune reaction stimulatory to their growth**.

## The case for the hypothesis in the mouse

### 1). The dose-response curve in mouse tumor immunity-

**Figure 1:**
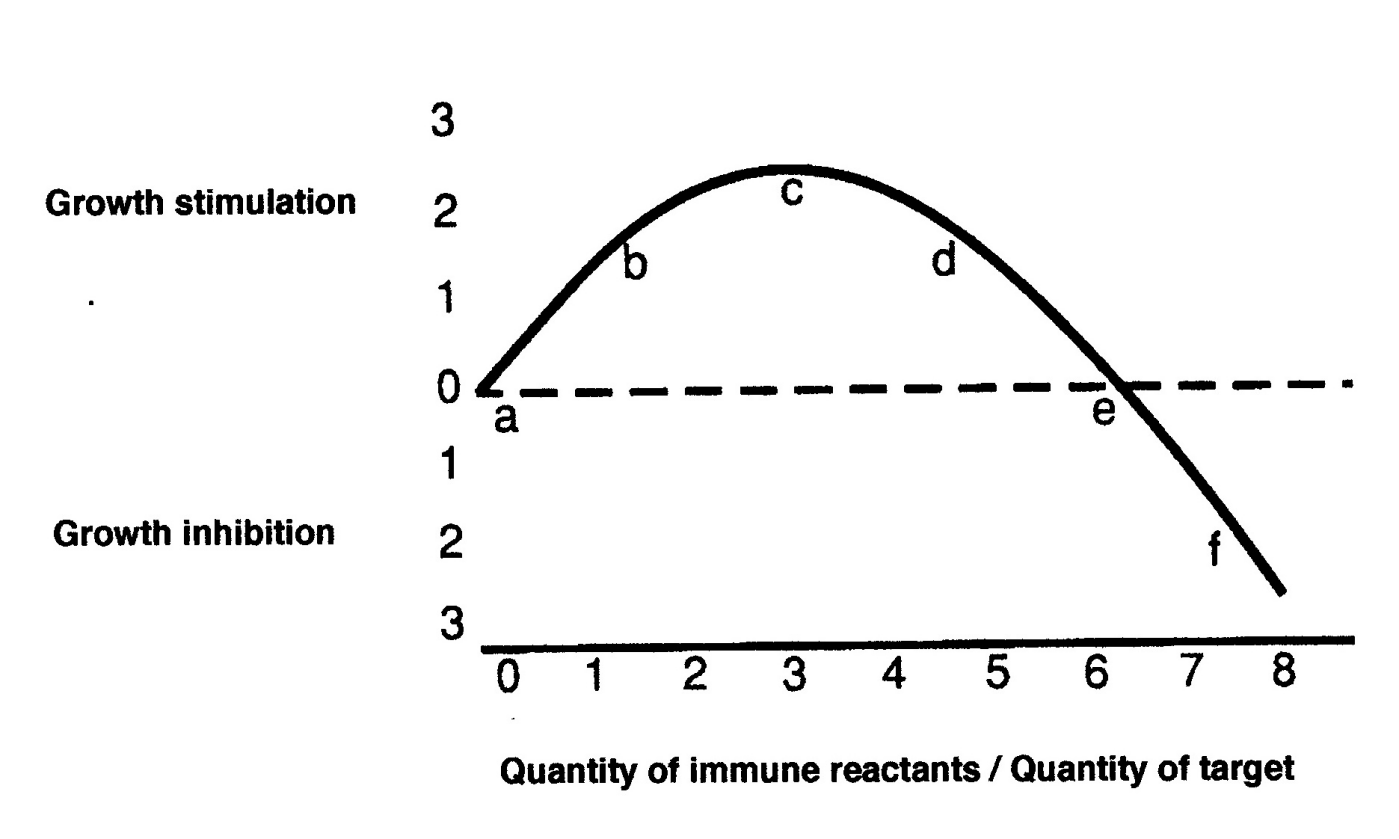
**Idealized immune reaction curve (IRC) of Winn test data from **[2]**relating the quantity of immune reactants to sarcoma growth**. The lettering and enumeration are arbitrary aids to discussion.

The immune response curve (IRC) relates the stimulation or inhibition of growth to the relative size of the specific immune reaction [Figure 1]. In general, a quantitatively weaker immunity has been shown to stimulate tumor growth while a larger quantity of apparently the same immune reactants may be inhibitory [2,3]. The idealised immune response curve (IRC) is largely based upon observations made with tumor antigens, but we think that it is probably also applicable to normal allo-antigens. The phenomenon of stimulated growth by a weak immune reaction was first suggested by the possibility that a weak immune reaction to a fetus may be a benefit to fetal survival [4]. Subsequently, the phenomenon of immunostimulation of growth was tested directly using Winn tests; a specific number of tumor cells was mixed with various numbers of syngeneic spleen cells harvested from mice that had either borne or not borne the specific tumor [2]. The tumor cell/spleen cell mixtures were inoculated s.c. into syngeneic mice that had, shortly before, been radiated and thymectomized in order to prevent, as far as possible, host contributions to any observed immunological effect. The results were clear [figure 1]. The relationship of the quantity of immune reactants to tumor growth was not linear; small proportions of immune spleen cells stimulated tumor growth while larger proportions were inhibitory [2].

Numerous other titrations, similar in principle, were performed by us as well as by others, both *in vitro *and *in vivo*, with similar results [3]. It seems particularly evident from titrations done with specific antibody and complement that one and the same immune reactant can probably be stimulatory or inhibitory depending only upon dosage [5]. A general review of the phenomenon of hormesis in relation to immune responses is to be found in [6].

In addition to the titrations, immunostimulation of tumor growth was explored indirectly by observations of other types. For example, in two large independent studies, each confirming the other, it was observed, in mouse carcinogenesis by 3-methylcholanthrene, that the tumors with the very shortest latencies had intermediate levels of immunogenicity. Thus, the most conducive immune-reaction for rapidity of tumor growth was not minimal; it was apparently a significantly positive, albeit low, reaction [7]. Other studies consistent with the immunostimulation hypothesis are outlined in [3].

### 2). Autochthonous tumor tolerance-

The paper that finally convinced most investigators that true anti-tumor immunity was actually possible in the mouse, showed the phenomenon by immunizing syngeneic mice with fragments of various 3-methylcholanthrene-induced sarcomas. After excision of the immunizing growth, the growths of subsequent challenge implants of the same tumor were evaluated [8]. The results were convincing because of controls that showed that the demonstrated immunity was specific for the tumor rather than for the mouse of origin. It was thus something of a puzzle that similar immunizations in the animal of origin failed to show immunity (unpublished personal observation). It remained for the Karolinska Institutet group to subsequently achieve some immunity in the very animal of origin by rather heroic repeated immunizations [9].

Later work, investigating this relative resistance to demonstrable immunization in the very animal in which the tumor had originated, showed that it was at least partially dependent upon the immunosuppression induced by the carcinogen [10]. However, relatively non-immunogenic mammary tumors, attributed to the mouse milk agent, grew better upon autochthonous transplantation than when transplanted to syngeneic animals [10]. In another study, challenge MCA-induced tumors grew better in the autochthonous mouse than did carcinogen-induced syngeneic tumors that were implanted into that same animal [11]. Both of these studies suggest a reduction of the immune response to stimulatory levels in the autochthonous host even in the absence of the action of a strong carcinogen.

Perhaps the first suggestion of a degree of tumor tolerance in the mouse of origin is to be found in a remarkably prescient paper published in 1916 (before the development of inbred mice); Fleisher and Loeb concluded that mice bearing *in situ *primary tumors were preferable soil for the growth of implants of allogenic, but previously untransplanted spontaneous tumors [12]. Such tumors usually failed to grow in normal control animals (since the animals were random-bred), but would grow as autografts. The growth of a primary spontaneous tumor apparently not only produced tolerance to tumor specific antigens, but additionally, tolerized the host to a variety of foreign histocompatibility antigens found in allogenic tumors. How could a primary tumor tolerize against a variety of such antigens? However, foreign histocompatibility antigens do exist in at least some tumors [13]. Thus, it appears that growth of an untransplanted primary tumor produces some tolerance in the primary host not only to the strain specific antigens, but also to a variety of histocompatibility antigens foreign to the host's adult phenotype, but apparently present nonetheless in the host's own tumor. The presence of these unexpected antigens could be the result of the thousands of mutations known to occur in cancers [14].

In addition to products of seemingly foreign genotypes, other data suggest that carcinogenesis may unveil antigenic moieties that played some role in the embryo, but that are unexpressed in the normal adult. In other words, a tumor is even more like an embryo than heretofore imagined, apparently reexpressing a spectrum of histocompatibility antigens that are hidden in the adult, but found in the tumor and embryo (carcino-embryonic antigens). This 1916 paper may be the first suggestion of onco-fetal antigens [15]. Perhaps the apparent rarity of cross reactions among MCA-induced tumors is due to the fact that immunity to onco-fetal antigens may often fall on the stimulatory part of the IRC, where they may sometimes be overlooked.

The 1916 paper that showed better growth of spontaneous tumors that were transplanted into mice already bearing a primary spontaneous tumor also showed a contrasting phenomenon; animals bearing a primary spontaneous tumor were poorer soil for the growth of a long transplanted tumor [12]. We suggest, as one possible explanation, that the long tranplanted tumor had evolved over many transplant generations to grow well in the face of a normal high level of allograft immunity; when this normal level was reduced by the tolerizing primary tumor the level of immunity fell to what were less stimulatory levels for the highly evolved long transplanted tumor.

At this point it must be noted that any tolerance produced by bearing a tumor is apparently confined to antigens associated with the tumor; responses to extraneous immunogens appear to be unaffected by the presence or absence of tumor [16].

### 3). Immunogenicity of spontaneous tumors-

As was already mentioned, spontaneous tumors, *ie*., tumors that occur sporadically without any known viral or other known etiological agent, have been proclaimed by some to be non-immunogenic. Hewitt was most vociferous [1]. Among a variety of observations, he reported testing seven sporadic spontaneous mouse tumors for signs of induced immunogenicity and could produce inhibition of tumor growth to none of them by immunization. However, in seven of the seven, the challenge tumor implants were actually stimulated when grown in the supposedly immunized animals [1]! Given the existence of the non linear IRC and the immunostimulation phenomenon, this is indeed the result that would be anticipated if the tumors had been, in actuality, weakly immunogenic. In our opinion, much rides upon whether this result by Hewitt, while statistically highly significant, is reproducible and general. All his other observations with sporadic tumors were not direct tests of immunization and all failed, in our estimation, to rule out the possibility of the existence of very weak and stimulatory immunogenicities.

We are also encouraged to think that sporadic spontaneous tumors may indeed have at least some degree of immunogenicity by the data of Fleisher and Loeb, which has already been discussed [12]. If spontaneous primary tumors induced a state of immune tolerance, as did at least some of the tumors these authors examined, surely the tumors must have been somewhat immunogenic. Admittedly, the histology of the tumors and the prevalence of the phenomenon are currently unknown.

### 4). Progression in animal tumors-

That immunity may promote dedifferentiation and progression has been suggested by a number of observations. Progression is commonly observed when tumors are transplanted serially in immunocompetent animals, but seems to be much delayed or lacking in immunodeprived hosts [17,18]. Some tumors may even become more differentiated when passaged in athymic nude mice [19]. Hammond passaged small-cell lung carcinomas from inbred hamsters into hamsters of differing immune capacities [20]. Since his paper may be difficult to obtain, we shall once again, as was done in a previous publication [3], quote directly from his conclusions: "....the rate and degree of dedifferentiation during tumor progression is directly related to the level of host immunocompetence. Immunodepression favors maintenance of the differentiated state, but normal or elevated immunoreactivity is associated with progressive dedifferentiation [20]." More recently, de Visser *et al*. have presented evidence for B-cell-dependent tumor progression [21] and Daniel *et al*. have shown that CD4+ T cells can enhance skin-cancer progression [22]. Thus, it seems probable, given that immunity can apparently stimulate progression among animal tumors, that immunity continues to stimulate growth in such tumors long after their inception and probably throughout their existence.

## The case for the new hypothesis in human tumors

### 1). The IRC in human cancer immunity-

As already discussed, mouse tumor immunity seems to be subject to nonlinear dosage effects; small dosages of anti-tumor immunity tend to stimulate tumor growth while larger dosages of the same immune reactants are likely to be inhibitory [3]. There is some evidence that human tumor immunity behaves in similar fashion.

Perhaps the most convincing evidence that human cancers may also exhibit a nonlinear immune reaction curve (IRC) in their response to immunity derives from the behavior of Kaposi's sarcoma, a viral tumor that is common in AIDS patients. The tumor commonly "flares" during the period of immune recovery while the AIDS is being treated, which suggests that the tumor grows best when the patients immune capacity is impaired, but not too impaired [23].

### 2). Autologous tolerance to human tumors-

Unfortunately, human cancers cannot be tested for immunogenicity by transplantation into immunized hosts as can mouse tumors. Consequently, we must rely upon less direct and less convincing evidence. The fact that human tumors, like mouse tumors, give little or no evidence of immunological progression despite progression in other parameters may imply, as seems to be true in the mouse, that partial tolerance to each new antigen probably keeps the putative immunogenicity low and in the stimulatory range.

### 3). Immunogenicity of spontaneous, episodic, human tumors-

The fact that immunodepression of humans does appear to alter somewhat the incidence of some types of tumor, usually resulting in an increased, but occasionally in a decreased incidence, is powerful, but indirect evidence, that immune mechanisms may play a role in sporadic, spontaneous human carcinogenesis [24-26], as well as it does in viral oncogenesis.

That immunity plays a similar role in virally induced tumors in the human and the mouse is suggested by the success of antiviral immunization in the prevention of human cervical cancer [27]. In our lab, Murasko showed that immunization of mice with small dosages of inactivated Moloney sarcoma virus enhanced the tumor producing effect of subsequent challenge with active virus while immunization with large dosages produced resistance to tumor production [28].

### 4). Progression in human tumors-

Biological progression, as distinct from mere enlargement, is a common attribute of human as well as animal cancers. As described by Foulds, it is characterized by focal areas of dedifferentiation probably attributable to new clonal characteristics in the involved tissue [29]. Thus, progression is probably associated with the repeated appearance of new antigens and is probably driven in man, as it seems to be in the mouse, by stimulatory immune reactions.

## The new hypothesis

### The concepts-

We suggest that what is usually called the immune system is a facet of a broader growth control mechanism that is concerned primarily with, and is an adjunct to, the regulation of the growth of normal organs and tissues especially in connection with differentiation and reproduction [30]. Levels of immune reactants to the immunogens of normal tissue are presumed to be, in the adult, at a level that produces neither stimulation nor inhibition of growth (equivalent to 'e' on the IRC), but may help to maintain the differentiated state. There is evidence suggesting that neoplastic tissue may, *in vivo*, be more sensitive than is normal tissue to the effects of changes in levels of immunity [31], perhaps only because tumor tissue is the smaller target *in vivo*. Thus, the IRC is probably displaced depending upon the characteristics of the tissues involved, but the dosage relationship is assumed to hold generally; *ie*., smaller quantities of immune reactants are more likely to be stimulatory while larger quantities of the same reactants tend to be more inhibitory.

Cancer cells are presumed to be endowed with one or more new immunogens. During progression we postulate that new immunogens arise frequently. The immune response to each new immunogen varies in magnitude, but each begins small and is therefore stimulatory; if not curtailed by specific tolerance, immunity to each such variant might eventually grow to an inhibitory level. However, most are curtailed by specific tolerance so the aggregate response usually remains in the stimulatory range, thus curtailing tumor remission.

Benign tumors, having experienced relatively little progression, presumably have fewer immunogens, and their progression awaits the arisal of new variant immunogenic cells. Growth stasis or dormancy suggests that the level of immunity to such a tumor, like that to normal self-cells, is at a point at which neither stimulation nor inhibition of growth occurs (equivalent to level 'e' on the IRC).

Systemic immunosuppression increases the frequency of a variety of skin cancers, but tends to decrease the incidence of breast and perhaps rectal tumors [24-26]. This suggests that, for reasons as yet obscure, a given level of systemic immunity has an amplified effect in the skin, but a diminished efficacy in breast and rectum. We offer the following possible explanation of the differential effect of immunodepression on skin and breast tumor incidences: there is evidence that a given level of immunity has more effect in some areas of the body than in others; in particular, the skin seems to be particularly potent at the efficient use of any particular level of systemic immunity [31]. We suggest that with normal levels of systemic immunity most skin tumors fall somewhere between 'd' and 'e' on the IRC and so are stimulated by moderate immunodepression, as is imposed naturally by aging or iatrogenically during organ allografting. In contrast, we postulate that with normal host immune capacity, breast tumors fall on the IRC near 'b' or 'c' and therefore immunodepression produces less tumor stimulation and thus a lowered tumor incidence.

There is some evidence that suggests that the longevity of benign tumors is influenced by the level of the immune reaction, minimal immunity being associated with shorter skin papilloma duration [32]. The longer the benign tumor's life, the greater the chance of the formation of further variants. The evidence that the host's immune capacity probably does affect the longevity of benign papillomas and hence their likelihood of transformation to malignancy has been discussed recently [32].

There is great confusion concerning the role of the immune system in cancer growth [fantastic review-33]. Much of this confusion undoubtedly arises from the complexities of the disease, but we feel that some of the confusion stems from a general lack of appreciation of the IRC; if the immune reaction can either stimulate or inhibit neoplastic growth depending only upon dosage relationships, only the most careful titrations are likely to have any chance of giving consistent and interpretable results.

## A new approach to immunotherapy

New variant cells are probably a major obstacle to cytotoxic therapies. Such cells produce, partly because of immunological tolerance as well as the fact that incipient immunity must necessarily be weak, tumor stimulating rather than inhibitory immune responses; thus, a decrease in the immune capacity of the host might inhibit the competitive advantage of newly arising variant cells. Some tumors might even become more differentiated if the general immune capacity of the tumor host were sufficiently reduced. If the immune stimulation, produced in reaction to new variant cells, could be held in check for long periods by drastically reducing the patient's systemic immune competency, tumor progression might be inhibited; some tumors might even become less malignant and cytotoxic therapies, already immunosuppressive, might be rendered more efficacious. Thus, the possible benefits of systemic immunosuppression must be weighed against the hazards such therapy would entail.

By contrast, attempting to raise the specific anti-tumor immunity in the face of autochthonus tumor tolerance, as by vaccination, is unlikely to produce permanent gains in most cases because of the frequent appearance of new immunologically stimulated antigenic variants associated with the processes of biological progression.

## Summary

The available evidence supports the hypothesis that **many (most?, all?) malignancies grow and progress, at least in part, because of persistent immunostimulation by weak immune responses**.

In view of the difficulties of overcoming autochthonous tumor tolerance, it might sometimes be more effective to attempt to inhibit a cancer by immunosuppression rather than by hyperimmunization.

## Competing interests

The authors declare that they have no competing interests.

## Authors' contributions

The authors contributed equally.

All authors read and approved the final manuscript.
